# Preconception folic acid supplementation for the prevention of birth defects: a prospective, population-based cohort study in mainland China

**DOI:** 10.1186/s12884-024-06283-8

**Published:** 2024-02-06

**Authors:** Qiongjie Zhou, Guiying Dong, Qiaomei Wang, Haiping Shen, Yiping Zhang, Shikun Zhang, Jingqi Chen, Xiaotian Li

**Affiliations:** 1 Department of Obsterics, Obstetrics and Gynecology Hospital of Fudan University, Shanghai, 200011 China; 2 Shanghai Key Laboratory of Female Reproductive Endocrine-Related Diseases, Shanghai, China; 3 Institute of Science and Technology for Brain-Inspired Intelligence, Fudan University, Shanghai, China; 4 National Research Institute for Family Planning, No. 12, Dahuisi Road, Haidian District, Beijing, China; 5 Department of Obsterics, Shenzhen Maternity and Child Healthcare Hospital, Shenzhen, 518028 Guangdong China

**Keywords:** Folic acid supplementation, Birth defects, Neural tubal defects, Clefts, congenital heart disease, China

## Abstract

**Background:**

Folic acid supplementation is recommended for reducing the risk of birth defects. We aimed to assess the protective association of periconception folic acid supplements with birth defects in real-world setting.

**Methods:**

This prospective, population-based cohort study utilized national preconception registered data of married Chinese couples planning a pregnancy within 6 months between 2010 and 2012 in Mainland China. Participated women are freely provided folic acid starting 3 months before conception till 3 months after conception. Birth defects were self-reported at 42 days postpartumn followup. R software (v4.0.2) was applied for statistical analyses.

**Results:**

Complete data of 567,547 couples with pregnancy outcomes and folic acid supplementation were extracted for final analysis. A total of 74.7% women were with folic acid supplementation, and 599 birth defects were self-reported. The odd of birth defects was lower among women taking folic acid compared to their counterparts not taking (0.102% vs 0.116%, *P* < 0.001). In the multiple logistic regression analyses, the odd of birth defects was lower among couples with maternal folic acid supplementation (OR = 0.78, 95%CI: 0.66–0.95, *P* = 0.011), especially decreased odd of neural tube defects (NTDs) (OR = 0.56, 95%CI: 0.39–0.82, *P* = 0.003). This association was confirmed by 1:4 and 1:10 case control analysis. Odds of birth defects were significantly lower among women with folic acid supplementation more than 3 months before pregnancy (*P* < 0.001), and moreover, the odds of cleft (*P* = 0.007) and NTDs (*P* = 0.007) were of notable decrease.

**Conclusion:**

This retrospective case cohort study provides programmatic evidence for public health strategy-making to for reducing the risk of NTDs and clefts.

**Supplementary Information:**

The online version contains supplementary material available at 10.1186/s12884-024-06283-8.

## Introduction

Periconception folic acid supplementation is a primary prevention intervention for reducing the risk of birth defects especially neural tube defects (NTDs) [1]. Early randomized controlled studies (RCTs) and cohort studies provided consistent beneficial evidence, which initiated from Hungarian randomized clinical trial in 1984, to cohort studies between 1984 and 1996 [2, 3]. Moreover, the US Preventive Service Task Force (USPSTF) recommended all women planning a pregnancy taking a daily dosage of 0.4–0.8 mg folic acid in 2009 [4–6]. However, the benefits of folic acid supplementation as a primary care are required to be informed.

In current postfortification era, there lacks new prospective studies to evaluate the benefits of folic acid supplement among women with pregnancy intention as a public health intervention [7]. Moreover, subsequent observational, case-control studies have not shown a protective association with birth defects such as NTDs [8]. These inconsistent protective association may be attenuated by greater controls for potential sources of bias, misclassification or recall bias. The potential for recall bias or difference by timing or duration of therapy, may attenuate the measured association. Despite of some known confounding including maternal age, education, smoking and alcohol consumption, other possible paternal factors should also be taken into consideration such as smoking and alcohol drinking. Additionally, placebo-controlled randomized trials that eliminate folic acid is challenging for the ethical consideration. Consequently, qualified prospective studies are scared to demonstrate a protective association of folic acid supplement among women with pregnancy intention as a real-world maternal health care.

Early randomized clinical and cohort studies provided consistent evidence of its benefit, but potentially offered greater controls for potential sources of bias, and ethical considerations for precluding placebo-controlled randomized trials that eliminate folic acid. Consequently, in the postfortification era, subsequent observational, case-control studies did not show a protective association with birth defects such as NTDs [8]. The potential for recall bias or difference by timing or duration of therapy, may attenuate the measured association. Additionally, despite of some known maternal confounding including age, education, occupation, smoking and alcohol consumption, these paternal factors should also be taken into consideration.

The Chinese government has launched the National Free Preconception Health Examination Project (NFPHEP) in 2010, in which, women planning a pregnancy within 6 months are freely provided take a daily 0.4 mg supplement of periconception folic acid. In this nation-wide public health care project, the timing and duration of folic acid supplementation, and pregnancy outcomes are followup. Nearly 74.7% women planning a pregnancy took folic acid supplemental before pregnancy [9, 10], and thus, this prospective cohort study provided an optimal opportunity for assessing the protective association of periconception folic acid supplements with NTDs, together with other birth defects, including clefts, congenital heart disease, limb anomalies, digestive tract anomalies, gastroschisis as well.

## Materials and methods

### Study design and participants

This prospective, population-based cohort study utilized NFPHEP database, which collected from couples intending to conceive within 6 months, covering 220 counties or districts across 31 provinces and province-level municipalities in mainland China during 2010–2012 [9, 10]. Married couples planning a pregnancy were freely provided with a preconception education and advocacy program on reproductive health by local family planning service agencies or maternal and children’s care service centers. Couples planning a pregnancy within 6 months with the wife’s age between 20 and 49 years, were included. Those women with incomplete information regarding folic acid supplementation or pregnancy outcomes, were excluded from the final analysis. This study followed the Strengthening and Reporting of Observational Studies in Epidemiology (STROBE) reporting guideline.

### Data collection

Specialized community healthcare staff inquired about the pregnancy intention of married couples residing in their community and provided them with preconception health examinations. Trained health care personnel conducted a face-to-face interview and medical examination. Enrolled women are freely provided 0.4 mg/d supplement of folic acid starting 3 months before conception till 3 months after conception. Their medical examination and follow-up data are uploaded and stored in the NFPHEP medical service information system. Detailed design, organization, and implementation of this project are described elsewhere [9, 10].

### Variables

The information of folic acid supplementation, socio-demographic and clinical information, including maternal and paternal age, education, occupation, residence status, exposure to harmful substances (smoking, toxic substances, noise, cats and dogs), maternal adverse pregnancy history (previous spontaneous abortion, fetal death, stillbirth, and preterm birth), wives with birth defects, were collected using standard questionnaires, and were extracted for analysis.

Information about maternal folic acid supplementation (taking/no taking), including its timing (≥3 months before conception, < 3 months before conception, after conception and no taking), was collected. The definitions of exposure to smoking, toxic substances, noise, cats and dogs, were categorized as yes and no. Passive smoking and alcohol consumption status was categorized as no, occasionally and often. The amount of alcohol consumed was not recorded, as Chinese people habitually consume alcohol from different containers and therefore the exact amount of alcohol ingested is difficult to record.

### Outcomes and measurements

The primary outcome was the incidence of birth defects. Birth defects were reported by parents or medical staff at 42 days postpartumn followup. Secondary outcomes were the top six types of birth defects, including congenital heart disease, limb anomalies (including syndactyly, polydactyly and congenital club foot), clefts (including cleft lip and cleft palate), digestive tract anomalies (including duodenal atresia, esophageal atresia stenosis, anorectal atresia, intestinal atresia, congenital diaphragmatic hernia, congenital megacolon and congenital intestinal obstruction), gastroschisis and neural tube defects (including spina bifida, anencephaly, congenital hydrocephalus, encephalocoele, hemicardiac malformation, cerebral haemangioma and brain dysplasia). Other types of birth defects were not included for subgroup analysis.

### Statistical analysis

We used numbers and proportions to describe the participants’ socio-demographic and clinical characteristics. Differences in baseline characteristics between with and without birth defects groups were examined using the χ^2^ test. To minimize bias, multiple logistic regression analysis and case-control analysis were conducted. Associated featured confounders including maternal and paternal age, education, occupation and residence, were adjusted. Matching was based upon maternal age, education, province, alcohol consumption and smoking behavior, and paternal alcohol consumption and smoking behavior (Table S1). Odds ratio (ORs) were adjusted by 31 associated featured confounders, and maternal and paternal age, education, occupation and residence. Statistical analyses were performed using R software (version 4.0.2; https://www.r-project.org). Two-sided *P*-values < 0.05 were considered statistically significant.

## Results

### Enrollment of participants

**Fig. 1:**
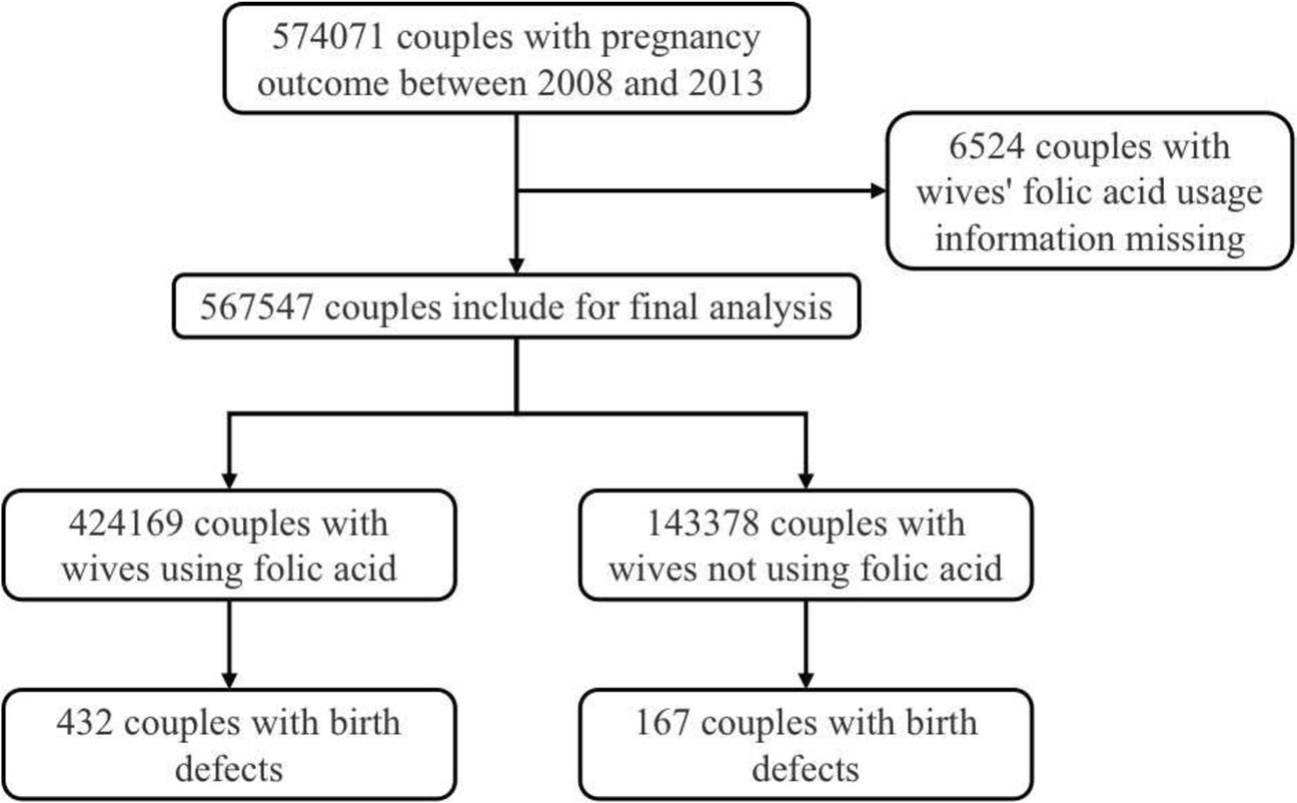
Enrollment of the participating couples

During the enrollment of the national preconception care project between 2010 and 2012, a total of 574,071 married couples intending to conceive with pregnancy outcomes were enrolled. Complete data of 567,547 couples with folic acid supplementation were extracted for final analysis, and data were missed in 6524 (1.1%) couples (Fig. 1). Baseline parameters between included and excluded couples were compared (Table S1).

Notably high portion of folic acid fortification among married women was observed (Table 1). In total, 424,169 (74.7%) were classified with folic acid supplementation (212,740 (37.5%) ≥3 months before conception, 83,765 (14.8%) < 3 months before conception, 127,232 (22.4%) after conception), and 143,378 (25.3%) with no folic acid intaking. Baseline parameters between couples with and without birth defects were compared (Table 1). Maternal adverse pregnancy history, anemia, using contraception, previous adverse pregnancy history, maternal and paternal exposure to passive smoking/toxic substances/noise/dogs and cats, vaginal bleeding/influenza/using drugs in early pregnancy were more frequent among couples with birth defects (*P* < 0.05).

**Table 1:** General parameters of total participated couples and case-controlled couples

	Total couples			Case-controlled couples (1:4)			Case-controlled couples (1:10)		
	No birth defects	Birth defects	*P* value	No birth defects	Birth defects	*P* value	No birth defects	Birth defects	*P* value
	(*N* = 566,948)	(*N* = 599)		(*N* = 2396)	(*N* = 599)		(*N* = 5990)	(*N* = 599)	
**Maternal age (years)**			0.094			0.706			0.833
20–24	139,837 (24.66)	142 (23.71)		540 (22.54)	142 (23.71)		1354 (22.60)	142 (23.71)	
25–29	282,041 (49.75)	300 (50.08)		1170 (48.83)	300 (50.08)		2930 (48.91)	300 (50.08)	
30–34	107,784 (19.01)	101 (16.86)		528 (22.04)	101 (16.86)		1307 (21.82)	101 (16.86)	
35–39	28,824 (5.08)	46 (7.68)		131 (5.47)	46 (7.68)		310 (5.18)	46 (7.68)	
≥ 40	8462 (1.49)	10 (1.67)		27 (1.13)	10 (1.67)		89 (1.49)	10 (1.67)	
**Paternal age (years)**			0.101			0.962			0.993
20–24	44,522 (7.85)	50 (8.35)		166 (6.93)	50 (8.35)		392 (6.54)	50 (8.35)	
25–29	283,375 (49.98)	276 (46.08)		1151 (48.04)	276 (46.08)		2892 (48.28)	276 (46.08)	
30–34	161,582 (28.50)	179 (29.88)		711 (29.67)	179 (29.88)		1794 (29.95)	179 (29.88)	
35–39	56,112 (9.90)	66 (11.02)		274 (11.44)	66 (11.02)		663 (11.07)	66 (11.02)	
≥ 40	21,357 (3.77)	28 (4.67)		94 (3.92)	28 (4.67)		249 (4.16)	28 (4.67)	
**Maternal education**			/						/
Illiteracy	1178 (0.21)	1 (0.17)	1.000	8 (0.33)	1 (0.17)	0.698	20 (0.33)	1 (0.17)	0.716
Primary school	24,503 (4.32)	32 (5.34)	0.226	127 (5.30)	32 (5.34)	1.000	318 (5.31)	32 (5.34)	0.924
Secondary school	368,259 (64.95)	390 (65.11)	0.966	1555 (64.90)	390 (65.11)	0.962	3893 (64.99)	390 (65.11)	0.964
High school	113,230 (19.97)	106 (17.70)	0.168	418 (17.45)	106 (17.70)	0.904	1043 (17.41)	106 (17.70)	0.865
College or undergraduate	58,952 (10.40)	70 (11.69)	0.315	283 (11.81)	70 (11.69)	1.000	701 (11.70)	70 (11.69)	1.000
Postgraduate or above	826 (0.15)	0 (0.00)	1.000	5 (0.21)	0 (0.00)	0.590	15 (0.25)	0 (0.00)	0.389
**Paternal education**			/			/			/
Illiteracy	558 (0.10)	1 (0.17)	0.446	8 (0.33)	1 (0.17)	0.698	14 (0.23)	1 (0.17)	1.000
Primary school	21,330 (3.76)	28 (4.67)	0.236	125 (5.22)	28 (4.67)	0.678	281 (4.69)	28 (4.67)	1.000
Secondary school	356,604 (62.90)	383 (63.94)	0.612	1495 (62.40)	383 (63.94)	0.509	3761 (62.79)	383 (63.94)	0.595
High school	122,920 (21.68)	109 (18.20)	0.042	465 (19.41)	109 (18.20)	0.524	1179 (19.68)	109 (18.20)	0.418
College or undergraduate	64,271 (11.34)	77 (12.85)	0.246	298 (12.44)	77 (12.85)	0.783	741 (12.37)	77 (12.85)	0.745
Postgraduate or above	1265 (0.22)	1 (0.17)	1.000	5 (0.21)	1 (0.17)	1.000	14 (0.23)	1 (0.17)	1.000
**Maternal occupation**			/			/			/
Farmers	432,297 (76.25)	444 (74.12)	0.230	1765 (73.66)	444 (74.12)	0.836	4444 (74.19)	444 (74.12)	0.961
Workers	56,391 (9.95)	54 (9.02)	0.494	242 (10.10)	54 (9.02)	0.445	615 (10.27)	54 (9.02)	0.357
Service officer	22,107 (3.90)	24 (4.01)	0.833	102 (4.26)	24 (4.01)	0.909	226 (3.77)	24 (4.01)	0.737
Business	11,154 (1.97)	13 (2.17)	0.658	46 (1.92)	13 (2.17)	0.742	108 (1.80)	13 (2.17)	0.522
Housewife	12,188 (2.15)	16 (2.67)	0.395	60 (2.50)	16 (2.67)	0.773	139 (2.32)	16 (2.67)	0.571
Teachers/officials	21,487 (3.79)	31 (5.18)	0.085	121 (5.05)	31 (5.18)	0.917	304 (5.08)	31 (5.18)	0.922
Others	11,324 (2.00)	17 (2.84)	0.142	60 (2.50)	17 (2.84)	0.665	154 (2.57)	17 (2.84)	0.685
**Paternal occupation**			/			/			/
Farmers	420,794 (74.22)	425 (70.95)	0.068	1719 (71.74)	425 (70.95)	0.723	4325 (72.2)	425 (70.95)	0.535
Workers	71,968 (12.69)	77 (12.85)	0.902	312 (13.02)	77 (12.85)	0.946	772 (12.89)	77 (12.85)	1.000
Service officer	19,821 (3.50)	19 (3.17)	0.823	97 (4.05)	19 (3.17)	0.346	217 (3.62)	19 (3.17)	0.645
Business	19,509 (3.44)	22 (3.67)	0.736	87 (3.63)	22 (3.67)	1.000	212 (3.54)	22 (3.67)	0.817
Househusband	778 (0.14)	0 (0.00)	1.000	6 (0.25)	0 (0.00)	0.606	13 (0.22)	0 (0.00)	0.623
Teachers/officials	20,316 (3.58)	35 (5.84)	0.006	98 (4.09)	35 (5.84)	0.075	259 (4.32)	35 (5.84)	0.096
Others	13,762 (2.43)	21 (3.51)	0.108	77 (3.21)	21 (3.51)	0.701	192 (3.21)	21 (3.51)	0.716
**Maternal residence status**			0.931			0.522			0.665
Rural	533,260 (94.06)	563 (93.99)		2232 (93.16)	563 (93.99)		5594 (93.39)	563 (93.99)	
Urban	33,688 (5.94)	36 (6.01)		164 (6.84)	36 (6.01)		396 (6.61)	36 (6.01)	
**Paternal residence status**			0.180			1.000			1.000
Rural	525,830 (92.75)	547 (91.32)		2186 (91.24)	547 (91.32)		5471 (91.34)	547 (91.32)	
Urban	41,118 (7.25)	52 (8.68)		210 (8.76)	52 (8.68)		519 (8.66)	52 (8.68)	
**Maternal adverse pregnancy history**			6.46e-5			0.469			0.631
Yes	15,244 (2.69)	34 (5.68)		119 (4.97)	34 (5.68)		312 (5.21)	34 (5.68)	
No	551,704 (97.31)	565 (94.32)		2277 (95.03)	565 (94.32)		5678 (94.79)	565 (94.32)	
**Wives with birth defects**			0.001			1.000			1.000
Yes	1070 (0.19)	6 (1.00)		27 (1.13)	6 (1.00)		69 (1.15)	6 (1.00)	
No	565,878 (99.81)	593 (99.00)		2369 (98.87)	593 (99.00)		5921 (98.85)	593 (99.00)	
**Maternal passive smoking**			/			/			/
No	474,858 (83.76)	461 (76.96)	1.83e-5	1833 (76.5)	461 (76.96)	0.829	4575 (76.38)	461 (76.96)	0.801
Occasionally	81,755 (14.42)	117 (19.53)	5.85e-4	482 (20.12)	117 (19.53)	0.775	1199 (20.02)	117 (19.53)	0.830
Often	10,335 (1.82)	21 (3.51)	0.005	81 (3.38)	21 (3.51)	0.900	216 (3.61)	21 (3.51)	1.000
**Maternal exposed to toxic substances**			5.96e-4			0.467			1.000
Yes	36,581 (6.45)	61 (10.18)		272 (11.35)	61 (10.18)		616 (10.28)	61 (10.18)	
No	530,367 (93.55)	538 (89.82)		2124 (88.65)	538 (89.82)		5374 (89.72)	538 (89.82)	
**Maternal exposed to noise**			5.76e-4			1.000			1.000
Yes	6952 (1.23)	18 (3.01)		74 (3.09)	18 (3.01)		188 (3.14)	18 (3.01)	
No	559,996 (98.77)	581 (96.99)		2322 (96.91)	581 (96.99)		5802 (96.86)	581 (96.99)	
**Maternal exposure to cats and dogs**			1.63e-4			0.640			0.745
Yes	10,964 (1.93)	26 (4.34)		93 (3.88)	26 (4.34)		244 (4.07)	26 (4.34)	
No	555,984 (98.07)	573 (95.66)		2303 (96.12)	573 (95.66)		5746 (95.93)	573 (95.66)	
**Paternal smoking**			3.04e-5			0.888			0.860
Yes	167,938 (29.62)	225 (37.56)		908 (37.90)	225 (37.56)		2275 (37.98)	225 (37.56)	
No	399,010 (70.38)	374 (62.44)		1488 (62.10)	374 (62.44)		3715 (62.02)	374 (62.44)	
**Paternal passive smoking**			/			/			/
No	401,575 (70.83)	375 (62.6)	1.51e-5	1487 (62.06)	375 (62.6)	0.814	3711 (61.95)	375 (62.6)	0.791
Occasionally	148,097 (26.12)	192 (32.05)	0.001	781 (32.6)	192 (32.05)	0.845	1926 (32.15)	192 (32.05)	1.000
Often	17,276 (3.05)	32 (5.34)	0.003	128 (5.34)	32 (5.34)	1.000	353 (5.89)	32 (5.34)	0.648
**Paternal alcohol consumption**			/			/			/
No	392,995 (69.32)	356 (59.43)	3.14e-7	1439 (60.06)	356 (59.43)	0.780	3549 (59.25)	356 (59.43)	0.965
Occasionally	166,814 (29.42)	233 (38.90)	7.38e-7	918 (38.31)	233 (38.9)	0.814	2340 (39.07)	233 (38.9)	0.965
Often	7139 (1.26)	10 (1.67)	0.355	39 (1.63)	10 (1.67)	1.0	101 (1.69)	10 (1.67)	1.0
**Paternal exposed to toxic substances**			5.0e-7			0.734			0.900
Yes	42,266 (7.46)	80 (13.36)		308 (12.85)	80 (13.36)		789 (13.17)	80 (13.36)	
No	524,682 (92.54)	519 (86.64)		2088 (87.15)	519 (86.64)		5201 (86.83)	519 (86.64)	
**Maternal folic acid supplementation**			0.145			0.039			0.024
Taking	423,737 (74.74)	432 (72.12)		1827 (76.25)	432 (72.12)		4573 (76.34)	432 (72.12)	
No taking	143,211 (25.26)	167 (27.88)		569 (23.75)	167 (27.88)		1417 (23.66)	167 (27.88)	
**Folic acid timing**									
≥ 3 months before conception	212,740 (37.52)	163 (27.21)	1.18e-7	852 (35.56)	163 (27.21)	1.11e-4	2116 (35.33)	163 (27.21)	5.92e-5
< 3 months before conception	83,765 (14.77)	97 (16.19)	0.327	426 (17.78)	97 (16.19)	0.400	1066 (17.8)	97 (16.19)	0.400
After conception	127,232 (22.44)	172 (28.71)	3.40e-4	549 (22.91)	172 (28.71)	0.003	1391 (23.22)	172 (28.71)	0.003
No taking	143,211 (25.26)	167 (27.88)	0.145	569 (23.75)	167 (27.88)	0.039	1417 (23.66)	167 (27.88)	0.024

Data were presented as mean (standard deviation)

Matching was based upon maternal age, educational level, province, alcohol consumption and smoking behavior, and paternal alcohol consumption and smoking behavior

### Association of folic acid supplementation with reduced odds of birth defects

A total of 599 birth defects were self-reported. Rate of birth defects was statistically discordant: it was lower among women taking folic acid compared to their counterparts not taking (0.102% vs 0.116%, *P* < 0.001) (Table 2). In the multiple logistic regression analyses, the odds of birth defects was lower among couples with maternal folic acid supplementation (OR = 0.78, 95%CI: 0.66–0.95, *P* = 0.011), especially that odds of NTDs (OR = 0.55, 95%CI: 0.39–0.82, *P* = 0.003). This association was confirmed by 1:4 and 1:10 case control analysis.

**Table 2:** Risk odds of birth defects related with maternal folic acid supplementation and based on different folic acid timing

	FA/NFA	Total couples		Case-controlled couples (1:4)		Case-controlled couples (1:10)	
		OR (95%CI)	*P* value	OR (95%CI)	*P* value	OR (95%CI)	*P* value
Folic acid supplementation*							
Total birth defects	432/167	0.78 (0.66,0.95)	0.011	0.81 (0.66,0.99)	0.044	0.81 (0.67,0.98)	0.033
Congenital heart disease	110/41	0.80 (0.55,1.16)	0.237	0.88 (0.59,1.33)	0.551	0.83 (0.57,1.22)	0.342
Limb anomalies	37/15	0.95 (0.50,1.80)	0.874	0.95 (0.46,1.96)	0.882	0.84 (0.44,1.58)	0.584
Clefts	73/33	0.72 (0.47,1.11)	0.136	0.66 (0.41,1.04)	0.075	0.69 (0.44,1.06)	0.086
Digestive tract anomalies	32/12	0.69 (0.36,1.32)	0.265	1.08 (0.53,2.23)	0.827	0.93 (0.46,1.88)	0.844
Neural tube defects	82/42	0.56 (0.39,0.82)	0.003	0.56 (0.37,0.86)	0.008	0.63 (0.42,0.93)	0.019
Gastroschisis	64/19	1.03 (0.61,1.75)	0.915	1.02 (0.57,1.83)	0.953	0.96 (0.56,1.66)	0.878
**Folic acid supplementation ≥ 3 months before pregnancy**							
Total birth defects	163/167	0.60 (0.48,0.75)	< 0.001	0.63 (0.49,0.80)	< 0.001	0.66 (0.53,0.83)	< 0.001
Congenital heart disease	42/41	0.63 (0.41,0.97)	0.036	0.73 (0.45,1.18)	0.197	0.73 (0.46,1.14)	0.167
Limb anomalies	12/15	0.51 (0.24,1.12)	0.093	0.69 (0.28,1.70)	0.415	0.61 (0.27,1.35)	0.220
Clefts	22/33	0.47 (0.27,0.82)	0.007	0.45 (0.25,0.82)	0.008	0.46 (0.26,0.80)	0.006
Digestive tract anomalies	13/12	0.62 (0.27,1.40)	0.251	0.90 (0.38,2.09)	0.798	0.80 (0.35,1.83)	0.597
Neural tube defects	38/42	0.55 (0.36,0.85)	0.007	0.51 (0.31,0.85)	0.009	0.61 (0.38,0.96)	0.034
Gastroschisis	30/19	0.97 (0.54,1.73)	0.913	0.99 (0.51,1.91)	0.966	0.94 (0.81,1.73)	0.833
**Folic acid supplementation < 3 months before pregnancy**							
Total birth defects	97/167	0.8**2** (0.64,1.06)	0.134	0.89 (0.67,1.18)	0.407	0.85 (0.65,1.11)	0.337
Congenital heart disease	24/41	0.65 (0.39,1.11)	0.115	0.88 (0.50,1.55)	0.652	0.76 (0.45,1.30)	0.321
Limb anomalies	10/15	0.94 (0.41,2.18)	0.888	0.98 (0.38,2.52)	0.961	0.97 (0.42,2.23)	0.934
Clefts	19/33	0.82 (0.46,1.48)	0.515	0.87 (0.45,1.67)	0.668	0.83 (0.46,1.50)	0.532
Digestive tract anomalies	8/12	0.84 (0.32,2.21)	0.720	1.21 (0.46,3.21)	0.701	1.09 (0.42,2.79)	0.865
Neural tube defects	16/42	0.43 (0.23,0.81)	0.008	0.56 (0.30,1.06)	0.076	0.58 (0.32,1.06)	0.075
Gastroschisis	97/167	0.75 (0.34,1.62)	0.463	1.13 (0.51,2.50)	0.755	1.04 (0.49,2.18)	0.924
**Folic acid supplementation after pregnancy**							
Total birth defects	172/167	1.02 (0.82,1.27)	0.870	1.06 (0.83,1.35)	0.671	1.02 (0.82,1.29)	0.838
Congenital heart disease	44/41	0.90 (0.58,1.39)	0.637	1.16 (0.71,1.91)	0.552	1.03 (0.66,1.63)	0.884
Limb anomalies	15/15	1.02 (0.50,2.11)	0.949	1.18 (0.51,2.71)	0.670	1.05 (0.50,2.24)	0.893
Clefts	32/33	0.97 (0.59,1.61)	0.909	0.84 (0.48,1.46)	0.531	0.93 (0.55,1.56)	0.782
Digestive tract anomalies	11/12	0.92 (0.39,2.17)	0.854	1.32 (0.53,3.28)	0.546	1.02 (0.43,2.39)	0.967
Neural tube defects	28/42	0.62 (0.38,1.01)	0.055	0.64 (0.37,1.10)	0.105	0.69 (0.42,1.15)	0.151
Gastroschisis	21/19	1.04 (0.55,1.96)	0.910	1.00 (0.50,2.00)	0.992	0.95 (0.49,1.83)	0.870

^*^:including folic acid supplementation ≥3 months before pregnancy, < 3 months before pregnancy and after pregnancy

*FA/NFA* folic acid usage / non folic acid usage. *OR* odd ratio, *CI* confidence interval

Matching was based upon maternal age, educational level, province, alcohol consumption and smoking behavior, and paternal alcohol consumption and smoking behavior

ORs were adjusted by maternal and paternal age, education, occupation and residence

### Effect of folic acid supplementation timing and dosage on reducing the odds of birth defects

Regarding folic acid timing, among women who took folic acid supplements ≥3 months months before conception, < 3 months before conception, after conception, and those who did not take the supplements, odds of birth defects were significantly lower among women with folic acid supplementation ≥3 months before pregnancy (OR = 0.60, 95%CI: 0.48–0.75, *P* < 0.001), together with lower odds of clefts (OR = 0.47, 95%CI: 0.27–0.82, *P* = 0.007) and NTDs (OR = 0.55, 95%CI: 0.36–0.85, *P* = 0.007), which was consistent in 1:4 and 1:10 case-control analysis (Table 2).

In addition, the effect of folic acid supplementation on birth defects were confirmed by comparing women who took before conception with those who didn’t take folic acid (OR = 0.68, 95%CI: 0.56–0.83, *P* < 0.001), while this effect was not statistically significant between women who took after conception with those who didn’t take folic acid (*P* > 0.05).

In addition, the odds of clefts was significantly decreased among couples with maternal folic acid supplementation before conception (OR = 0.59, 95%CI: 0.36–0.95, *P* = 0.029) and this odd was of no difference when comparing women who took after conception with those who didn’t take folic acid (*P* > 0.05) (Table S2).

## Comment

### Principle findings

In the postfortification era, the benefits of preconceptional folic acid supplementation on reducing the odds of birth defects require comprehensive consideration before implementation. This study is one of the first prospective cohorts providing essential, real-world evidence that, preconceptional folic acid supplementation of 0.4 mg/d dosage is protective for reducing the odds of offspring’s birth defects. With the great maternal health investment from Chinese government, periconceptional folic acid intake has achieved a high coverage among women with pregnancy intention [9]. Our findings are important for continuous efforts for promoting this public health policy for reducing the risk of some birth defects such as NTDs and clefts.

### Results

In this large programmatic evaluation of periconception folic acid supplementation strategy, the comparative effectiveness on reducing the risk of birth defect was estimated in 574,071 married couples in Mainland China. We found that wives who in-took folic acid before conception had lower odds of cleft and NTDs. Folic acid supplementation might substantially decrease the risk of some types of common birth defects, especially with regular and full-term supplementation at least 3 months before conception and in the early 3 months of pregnancy.

### Clinical implications

Our findings support periconception folic acid supplementation as an essential component of preconception care and indicate that folic acid might be beneficial for reducing the odds of NTDs and cleft, which is in agreement with evidence from previous randomized trials and cohorts [11–16]. These findings might be useful for maternal health care considering implementation of folic acid supplementation to reduce the risk of birth defects. Further studies are required to elucidate whether folic acid supplements be used consistently over the periods demarcated and whether our women be asked to take the supplements throughout pregnancy or only up to a certain point.

### Research implications

Second, we found that folic acid supplementation of 0.4 mg/d dosage before pregnancy appeared to be protective for reducing the risks of offspring’s birth defects including clefts and NTDs. Due to concerns about potential adverse effects, this lower dosage is commonly recommended in many countries including high-income countries, which was much lower than in the previous RCTs for NTDs [2, 3]. However, the key question should be addressed is the efficacy of this low dosage has not been well tested in previous randomized or cohort studies [17, 18]. Therefore, our study provided real-world evidence for evaluating the efficacy of the 0.4 mg/d dosage.

### Strengths and limitations

The main strength of this study is the use of data from the Chinese nation-wide preconception care database, characterized by high folic acid supplementation rate, adjusted associated maternal and paternal parameters, and matched known possible interfering factors, gave the power to detect subtle effect of folic acid supplementation. First, our dataset is reliable since it is based on prospective enrollment and nationwide coverage, with more than 85% coverage of couples intending to conceive and less than 10% missing data rate. Second, our study sample is vigorous, which has enrolled more than 2 million couples spanning 220 rural areas across all 31 provinces in mainland China. Third, this Chinese preconception health care strategy providing free folic acid supplementation seems feasible and well-implemented, assuring nearly 75% folic acid supplementation rate, compared to approximately 30–50% reported in the United States and some European countries [19], which was relatively lower than that in this study. Fourth, our protective association of folic acid supplementation with reducing birth defects was analyzed by conditional logistic regression and case-control analysis to minimize bias. A total of 31 associated featured items, together with some recognized confounders (including maternal and paternal age, education, occupation and residence), were adjusted.

Our study had some limitations. First, a definite causal relationship cannot be inferred from the cohort design, and a well-designed randomized study to evaluate folic acid fortification on reducing the risk of birth defects is difficult due to ethical considerations. Nevertheless, this prospective cohort seems more feasible, and the database used in the present study is ideal for analyzing their association. Second, there was a possible recall bias of the dosage and duration of folic acid supplementation and the prevalence of birth defects may be underestimated, considering that these pregnancy related information was based on questionnaires. To account for confounding effect, conditional logistic regression analysis featured with associated factors was simultaneously conducted to minimize bias. Third,

Our outcomes of birth defects were based on self-report at 42 days, which could lead to substantial misclassification as well as missing some birth defects. We have conducted 1:4 and 1:10 case-control analysis were simultaneously conducted to minimize bias, and the results of reduced risk association were confirmed.

### Conclusions

In summary, given the priority of periconception folic acid supplementation, our study provides programmatic evidence for public health strategy-making to improve offspring’s birth outcomes and life quality.

### Supplementary Information

**Additional file 1.**

## Data Availability

The datasets during the current study are available from the corresponding author on reasonable request.

## Abbreviations

NPHCP: the National preconception health care project
NTDs: Neural tube defects
ORs: Odds ratio
RCT: Randomized controlled study
STROBE: the Strengthening and reporting of observational studies in epidemiology
USPSTF: the US preventive service task force
