# Supplementary material for: Preconception folic acid supplementation for the prevention of birth defects: a prospective, population-based cohort study in mainland China
Source: BMC Pregnancy Childbirth. 2024 Feb 6;24:114. doi: 10.1186/s12884-024-06283-8 (PMC10845381; doi:10.1186/s12884-024-06283-8)
Supplement: Supplementary file 1 — Additional file 1. [file 12884_2024_6283_MOESM1_ESM.docx]

**Preconception folic acid supplementation for the prevention of birth defects: a prospective, population-based cohort study in mainland China**

Table S1. Baseline characteristics comparison between included and excluded couples.

Table S2. Provincial characteristics of total participants and case control groups.

Table S3. Risk odds of birth defects related with maternal folic acid supplementation before and after conception.

Table S1. Baseline characteristics comparison between included and excluded couples.

|  |  | Included | Excluded |  |
| --- | --- | --- | --- | --- |
|  |  | (N=567547) | (N=1575356) | P value |
| Maternal age (years) |  |  |  | 0 |
|  | 20-24 | 139979 (24.66) | 367224 (23.31) |  |
|  | 25-29 | 282341 (49.75) | 697535 (44.28) |  |
|  | 30-34 | 107885 (19.01) | 322931 (20.5) |  |
|  | 35-39 | 28870 (5.09) | 122265 (7.76) |  |
|  | ≥40 | 8472 (1.49) | 65401 (4.15) |  |
| Paternal age (years) |  |  |  | 0 |
|  | 20-24 | 44572 (7.85) | 162067 (10.29) |  |
|  | 25-29 | 283651 (49.98) | 657481 (41.74) |  |
|  | 30-34 | 161761 (28.5) | 443725 (28.17) |  |
|  | 35-39 | 56178 (9.9) | 194527 (12.35) |  |
|  | ≥40 | 21385 (3.77) | 117556 (7.46) |  |
| Maternal education |  |  |  | 0 |
|  | Illiteracy | 1179 (0.21) | 4188 (0.27) |  |
|  | Primary school | 24535 (4.32) | 75140 (4.77) |  |
|  | Secondary school | 368649 (64.95) | 1050642 (66.69) |  |
|  | High school | 113336 (19.97) | 274821 (17.45) |  |
|  | College or undergraduate | 59022 (10.4) | 166493 (10.57) |  |
|  | Postgraduate or above | 826 (0.15) | 4072 (0.26) |  |
| Paternal education |  |  |  | 0 |
|  | Illiteracy | 559 (0.1) | 2159 (0.14) |  |
|  | Primary school | 21358 (3.76) | 66310 (4.21) |  |
|  | Secondary school | 356987 (62.9) | 1030066 (65.39) |  |
|  | High school | 123029 (21.68) | 294105 (18.67) |  |
|  | College or undergraduate | 64348 (11.34) | 177460 (11.26) |  |
|  | Postgraduate or above | 1266 (0.22) | 5256 (0.33) |  |
| Maternal occupation |  |  |  | 0 |
|  | Farmers | 432741 (76.25) | 1219423 (77.41) |  |
|  | Workers | 56445 (9.95) | 131090 (8.32) |  |
|  | Service | 22131 (3.9) | 67745 (4.3) |  |
|  | Business | 11167 (1.97) | 30378 (1.93) |  |
|  | Housewife | 12204 (2.15) | 27328 (1.73) |  |
|  | Teachers/officials/ | 21518 (3.79) | 70253 (4.46) |  |
|  | Others | 11341 (2.0) | 29139 (1.85) |  |
| Paternal occupation |  |  |  | 0 |
|  | Farmers | 421219 (74.22) | 1196372 (75.94) |  |
|  | Workers | 72045 (12.69) | 167184 (10.61) |  |
|  | Service | 19840 (3.5) | 59549 (3.78) |  |
|  | Business | 19531 (3.44) | 47622 (3.02) |  |
|  | Housewife | 778 (0.14) | 2059 (0.13) |  |
|  | Teachers/officials/ | 20351 (3.59) | 65926 (4.18) |  |
|  | Others | 13783 (2.43) | 36644 (2.33) |  |
| Maternal residence status |  |  |  | 3.78e-95 |
|  | Rural | 533823 (94.06) | 1469281 (93.27) |  |
|  | Urban | 33724 (5.94) | 106075 (6.73) |  |
| Paternal residence status |  |  |  | 4.92e-70 |
|  | Rural | 526377 (92.75) | 1449511 (92.01) |  |
|  | Urban | 41170 (7.25) | 125845 (7.99) |  |

Data were presented as mean (standard deviation).

Table S2. Provincial characteristics of total participants and case control groups.

|  | Total participants | | | Case control group (1:4) | | | Case control group (1:10) | | |
| --- | --- | --- | --- | --- | --- | --- | --- | --- | --- |
|  | No birth defects | Birth defects | Total | No birth defects | Birth defects | Total | No birth defects | Birth defects | Total |
| Anhui | 14103 | 23 | 14126 | 62 | 23 | 85 | 159 | 23 | 182 |
| Beijing | 1021 | 4 | 1025 | 6 | 4 | 10 | 24 | 4 | 28 |
| Chongqing | 14468 | 16 | 14484 | 55 | 16 | 71 | 132 | 16 | 148 |
| Fujian | 4611 | 6 | 4617 | 33 | 6 | 39 | 62 | 6 | 68 |
| Gansu | 9310 | 10 | 9320 | 37 | 10 | 47 | 95 | 10 | 105 |
| Guangdong | 88493 | 83 | 88576 | 355 | 83 | 438 | 906 | 83 | 989 |
| Guangxi | 16418 | 29 | 16447 | 115 | 29 | 144 | 310 | 29 | 339 |
| Guizhou | 6085 | 26 | 6111 | 101 | 26 | 127 | 253 | 26 | 279 |
| Hainan | 757 | 4 | 761 | 16 | 4 | 20 | 39 | 4 | 43 |
| Hebei | 48521 | 95 | 48616 | 381 | 95 | 476 | 915 | 95 | 1010 |
| Heilongjiang | 756 | 0 | 756 | 0 | 0 | 0 | 4 | 0 | 4 |
| Henan | 63478 | 47 | 63525 | 192 | 47 | 239 | 498 | 47 | 545 |
| Hubei | 77539 | 64 | 77603 | 283 | 64 | 347 | 693 | 64 | 757 |
| Hunan | 71301 | 41 | 71342 | 142 | 41 | 183 | 391 | 41 | 432 |
| Jiangsu | 28733 | 29 | 28762 | 104 | 29 | 133 | 253 | 29 | 282 |
| Jiangxi | 6917 | 8 | 6925 | 28 | 8 | 36 | 71 | 8 | 79 |
| Jilin | 17293 | 2 | 17295 | 1 | 2 | 3 | 9 | 2 | 11 |
| Liaoning | 1191 | 1 | 1192 | 9 | 1 | 10 | 23 | 1 | 24 |
| Neimenggu | 1283 | 1 | 1284 | 2 | 1 | 3 | 11 | 1 | 12 |
| Ningxia | 1030 | 3 | 1033 | 6 | 3 | 9 | 12 | 3 | 15 |
| Qinghai | 333 | 0 | 333 | 0 | 0 | 0 | 1 | 0 | 1 |
| Shandong | 16039 | 27 | 16066 | 89 | 27 | 116 | 206 | 27 | 233 |
| Shanghai | 86 | 0 | 86 | 0 | 0 | 0 | 0 | 0 | 0 |
| Shannxi | 20647 | 33 | 20680 | 53 | 33 | 86 | 162 | 33 | 195 |
| Shanxi | 3364 | 9 | 3373 | 37 | 9 | 46 | 95 | 9 | 104 |
| Sichuan | 20982 | 11 | 20993 | 81 | 11 | 92 | 194 | 11 | 205 |
| Tianjin | 1285 | 0 | 1285 | 4 | 0 | 4 | 11 | 0 | 11 |
| Xinjiang | 5965 | 1 | 5966 | 39 | 1 | 40 | 69 | 1 | 70 |
| Yunnan | 18818 | 11 | 18829 | 99 | 11 | 110 | 240 | 11 | 251 |
| Zhejiang | 6121 | 15 | 6136 | 66 | 15 | 81 | 152 | 15 | 167 |

Data were presented as number.

Table S3. Risk odds of birth defects related with maternal folic acid supplementation before and after conception.

|  | FA/NFA | Total couples | | Case control couples (1:4) | | Case control couples (1:10) | |
| --- | --- | --- | --- | --- | --- | --- | --- |
|  |  | OR (95%CI) | P value | OR (95%CI) | P value | OR (95%CI) | P value |
| **Folic acid supplementation before pregnancy^*^** | | | | | | | |
| Total birth defects | 260/167 | 0.68 (0.56,0.83) | <0.001 | 0.70 (0.56,0.88) | 0.002 | 0.72 (0.58,0.88) | 0.002 |
| Congenital heart disease | 66/41 | 0.72 (0.48,1.07) | 0.103 | 0.78 (0.50,1.20) | 0.254 | 0.74 (0.49,1.12) | 0.151 |
| Limb anomalies | 22/15 | 0.69 (0.36,1.32) | 0.265 | 0.80 (0.36,1.79) | 0.583 | 0.73 (0.37,1.47) | 0.381 |
| Clefts | 41/33 | 0.59 (0.36,0.95) | 0.029 | 0.57 (0.45,1.14) | 0.033 | 0.57 (0.36,0.93) | 0.023 |
| Digestive tract anomalies | 21/12 | 0.89 (0.41,1.92) | 0.756 | 1.00 (0.46,2.15) | 0.994 | 0.89 (0.42,1.88) | 0.760 |
| Neural tube defects | 54/42 | 0.59 (0.39,0.89) | 0.012 | 0.53 (0.36,0.83) | 0.006 | 0.60 (0.39,0.91) | 0.017 |
| Gastroschisis | 43/19 | 1.09 (0.63,1.91) | 0.752 | 1.03 (0.55,1.92) | 0.002 | 0.97 (0.54,1.72) | 0.903 |
| **Folic acid supplementation after pregnancy** | | | | | | | |
| Total birth defects | 172/167 | 1.00 (0.80,1.25) | 0.991 | 1.05 (0.82,1.34) | 0.686 | 1.02 (0.81,1.28) | 0.861 |
| Congenital heart disease | 44/41 | 1.04 (0.67,1.60) | 0.877 | 1.16 (0.71,1.91) | 0.558 | 1.03 (0.66,1.63) | 0.887 |
| Limb anomalies | 15/15 | 1.02 (0.50,2.07) | 0.958 | 1.18 (0.51,2.70) | 0.702 | 1.05 (0.49,2.23) | 0.900 |
| Clefts | 32/33 | 1.05 (0.63,1.75) | 0.850 | 0.81 (0.47,1.42) | 0.465 | 0.92 (0.55,1.55) | 0.763 |
| Digestive tract anomalies | 11/12 | 1.14 (0.47,2.75) | 0.767 | 1.32 (0.53,3.28) | 0.546 | 1.02 (0.43,2.40) | 0.963 |
| Neural tube defects | 28/42 | 0.73 (0.45,1.18) | 0.203 | 0.64 (0.37,1.10) | 0.106 | 0.69 (0.42,1.15) | 0.152 |
| Gastroschisis | 21/19 | 1.24 (0.66,2.33) | 0.508 | 1.00 (0.50,2.00) | 0.993 | 0.95 (0.49,1.82) | 0.865 |

^*^：including folic acid supplementation ≥3 months before pregnancy and <3 months before pregnancy.

FA/NFA: folic acid usage / non folic acid usage. OR: odd ratio; CI: confidence interval.

Matching was based upon maternal age, educational level, province, alcohol consumption and smoking behavior, and paternal alcohol consumption and smoking behavior.

ORs were adjusted by 31 associated featured confounders, and maternal and paternal age, education, occupation and residence.
